# LncRNA NEAT1 Promotes Inflammatory Response in Sepsis via the miR-31-5p/POU2F1 Axis

**DOI:** 10.1007/s10753-021-01436-9

**Published:** 2021-03-12

**Authors:** Yang Yang, Jianhua Xue, Lili Qin, Jiaxuan Zhang, Jiajia Liu, Junbo Yu

**Affiliations:** 1 Department of Trauma Center, Affiliated Hospital of Nantong University, No. 20, Xisi Road, Chongchuan District, Nantong, 226001 Jiangsu Province China; 2 Department of Endoscopic Center, Affiliated Hospital of Nantong University, Nantong, 226001 Jiangsu Province China

**Keywords:** sepsis, inflammatory response, NEAT1, miR-31-5p, POU2F1

## Abstract

Sepsis is considered to be a systemic inflammatory response, which results in organ dysfunction. LncRNA nuclear-enriched abundant transcript 1 (NEAT1) involved in sepsis progression has been reported. However, the underlying mechanism of NEAT1 in sepsis-induced inflammatory response remains to be revealed. In this study, NEAT1 and POU domain class 2 transcription factor 1 (POU2F1) were highly expressed in LPS-induced septic RAW264.7 cells, opposite to miR-31-5p expression. Furthermore, we found that NEAT1 silencing inhibited LPS-induced inflammatory response and cell proliferation, and promoted cell apoptosis. Subsequently, we found that miR-31-5p interacted with NEAT1 and targeted the 3′UTR of POU2F1, and in LPS-induced RAW264.7 cells, the inhibition of NEAT1 silencing was reversed by miR-31-5p knockdown, while POU2F1 downregulation could cover the functions of miR-31-5p knockdown. In a word, this study indicates that NEAT1 inhibits the LPS-induced progression of sepsis in RAW264.7 cells by modulating miR-31-5p/POU2F1 axis, suggesting that NEAT1 will be the potential therapeutic target for sepsis.

## INTRODUCTION

Sepsis is a systemic inflammatory response syndrome (SIRS) resulting from infection [1], which often occurs in patients with severe burns, trauma, and surgery. It manifests as shortness of breath, tachycardia, hemolytic anemia, fever or hypothermia, changes in platelet or white blood cell count, tissue damage, septic shock. Eventually, it develops into multiple organ dysfunction syndrome or multiple system organ failure [2–4]. Despite continued improvements in the mechanisms and treatment of sepsis in recent decades, the incidence and mortality of sepsis remain increasing due to the population ages [5, 6]. Recent epidemiological data show that the incidence of sepsis is about 0.45% [7], far exceeding myocardial infarction [8], the sum of prostate cancer, breast cancer, and lung cancer [9]. It is estimated that more than 18 million cases of sepsis occur in the world annually and their overall fatality rate is between 30 and 50% [10, 11]. At present, the pathogenesis of sepsis is not clear, so it has become an urgent task to explore the pathogenesis of sepsis and find new ways of prevention and treatment.

Long non-coding RNA (lncRNA) is a non-coding RNA (ncRNA) with a length of more than 200 base pairs [12]. A great deal of research shows that lncRNA plays an important role in biological processes, including cellular immunity, mitochondrial dysfunction, and apoptosis, and has gradually become a biomarker for various diseases, including sepsis [13]. For example, lncRNA PVT1 induces an increase in TNF-α, IL-6, and IL-1β release, and promotes inflammation by regulating TNF-α and JNK/NF-κB signaling pathways in sepsis [14]. Fang *et al.* [15] find that lncRNA H19 reduces the expression of miR-874, downregulates the secretion of inflammatory factors, and restores LPS-induced inflammatory response disorder and myocardial dysfunction in sepsis mice. NEAT1 in circulating blood is associated with increased disease risk, ascending severity of the disease, poor prognosis, and rising expression of inflammatory factors in sepsis patients [16], and Chen *et al*.’s study shows that NEAT1 is highly expressing in patients with sepsis, and aggravates LPS-induced cell injury via regulating miR-204/NF-κB axis [17], suggesting that NEAT1 has a significant pro-inflammatory effect. Although these studies suggest that NEAT1 is closely related to sepsis-induced inflammatory response, the specific mechanism of NEAT1 in sepsis progression is still worth investigating.

In our research, we explore the function and potential underlying mechanism of NEAT1 in sepsis-induced inflammatory response. Firstly, NEAT1 and POU2F1, along with the levels of inflammatory factors, including TNF-α, IL-6, and IL-1β, were increased in LPS-induced septic RAW264.7 cells, while miR-31-5p was decreased. In addition, NEAT1 interacts with miR-31-5p, targeting POU2F1 to modulate the LPS-induced inflammatory response, the proliferation, and apoptosis in RAW264.7 cells. Our study provided a potential target for sepsis-induced inflammatory response.

## MATERIALS AND METHODS

### Cell Culture

RAW264.7 cells were obtained from ATCC (Rockville, MD, USA), and cultured with DMEM medium containing 10% fetal bovine serum (FBS, Excel), 1% penicillin/streptomycin at 37 °C with 5% CO_2_. All experiments used the cell between passages 2 and 5.

### Cell Transfection

Short hairpin RNA targeting NEAT1 and the negative control were purchased from Ribobio Co., Ltd. (Guangzhou, China). Sh-NEAT1 (100 nM) was transient transfected using Lipofectamine 3000 (Thermo Fisher, CA, USA) according to the manufacturer’s instructions. The shRNA negative control was used as the control. After transfection 48 h, transfected cells were collected for subsequent analysis and detection.

### qRT-PCR

Total RNA was separated from sample through GenElute™ Total RNA Purification Kit (Merck, Darmstadt, DEU) according to the protocol. Then, RNA was reverse-transcribed into cDNA using QuantiTect Reverse Transcription Kit (Qiagen, Dusseldorf, DEU) on the instrument of Rotor-Gene Q 2plex (Qiagen). Next, Rotor-Gene ^TM^ SYBR Green PCR Kit (Qiagen) was used to do qRT-PCR and detected by on Rotor-Gene Q 2plex. Primers used for amplification were shown in Table 1.

**Table 1:** Primers used for amplification of qRT-PCR

Gene	Forward primer (5′–3′)	Reverse primer (5′–3′)
Lnc NEAT1	GTAATTTTCGCTCGGCCTGG	ACCCGAGACTACTTCCCCA
TNF-α	CCCTCACACTCAGATCATCTTCT	GCTACGACGTGGGCTACAG
IL-6	TAGTCCTTCCTACCCCAATTTCC	TTGGTCCTTAGCCACTCCTTC
IL-1β	GAAATGCCACCTTTTGACAGTG	TGGATGCTCTCATCAGGACAG
miR-31-5p	GCGCAGGCAAGATGCTCG	GTGCAGGGTCCGAGGT
POU2F1	AGCTGGGACAAGTTTACAGGC	TCCCGACTCTTCACTGGATTTA
β-actin	GCACCACACCTTCTACAATG	TGCTTGCTGATCCACATCTG
U6	TCCGGGTGATGCTTTTCCTAG	CGCTTCACGAATTTGCGTGTCAT

### ELISA Assay

TNF-α, IL-6, and IL-1β ELISA kits (Mibio, Shanghai, China) were used to measure the concentration of TNF-α, IL-6, and IL-1β following the instructions, and detected the absorbance at 450 nm by automatic microplate reader (BioTek, USA).

### CCK-8 Assay

The cell viability of RAW264.7 cells was assessed by CCK-8 assay. Firstly, the cells were inoculated onto 96-well plates and incubated at 37 °C, 5% CO_2_. Then, 10 μL CCK-8 solution was added into each hole and incubated for 1 h. Finally, the absorbance at 450 nm was determined by a microplate reader.

### EDU Staining

The cell proliferation was measured by EDU kit (Ribobio, Guangzhou, China). Briefly, logarithmic phase cells were placed into 96-well plates at 4 × 10^3^~1 × 10^5^/well. Then, 100 μL EDU solution was added into each well for 2 h. After washed with PBS, 4% paraformaldehyde was used to fix the cells for 30 min, and stimulated with glycine for 5–10 min. Eventually, the cells were incubated with 1X Apollo® solution in room temperature for 30 min, and observed under a fluorescence microscope.

### Western Blot Analysis

The sample was lysed by RIPA containing PMSF. Total protein concentration was detected by a BCA protein assay kit (Yeasen, Shanghai, China). Forty micrograms of proteins was separated by sodium dodecyl sulfate polyacrylamide gel electrophoresis (SDS-PAGE), and transferred into PVDF membranes followed by blocked with skim milk. Next, the membranes were incubated with primary antibodies (PCNA, Ki-67, Bax, Bcl-2, cleave-Caspase 3, cleave-Caspase 9, and β-actin) overnight at 4 °C. Then, goat anti-rabbit secondary antibody was used to incubate membranes for 1 h. In the end, these bands were measured by enhanced chemiluminescence (Yeasen, Shanghai, China) and quantified by densitometry. The antibodies PCNA (1:1000, ab265609), Ki-67 (1:1000, ab205718), Bax (1:500, ab32503), Bcl-2 (1:1000, 32124), cleave-Caspase 3 (1:500, ab32042), cleave-Caspase 3 (1:500, ab2324), and β-actin (1:1000, ab8227) were purchased from Abcam (Cambridge, UK).

### TUNEL Staining

The cell apoptosis was detected by TUNEL staining. Firstly, cells were inoculated on 96-well plate until reaching approximately 80% confluence. Then, 4% paraformaldehyde fixed cells for 30 min, and then the cells were permeated in 0.3% Triton X-100 for 2 h at room temperature. After PBS washing, 0.3% H_2_O_2_ was adopted to inactivate the endogenous peroxidase. Then, the cells were incubated in TUNEL solution at 37 °C in dark for 60 min followed by PBS washing. Next, the cells were incubated with DAB staining solution at room temperature for 5–30 min. In the end, after stained with DAPI, the cells were observed and photographed under fluorescence microscope, and analyzed with ImageJ.

### Luciferase Reporter Assay

Dual-Luciferase Gene Reporter Assays was previously described [18]. In brief, the wild-type (WT) and mutant (Mut) miR-31-5p binding site sequences in the NEAT1 3′-untranslated region were used to generate NEAT1-WT vector and NEAT1-Mut vector using pGL3 as backbone (Promega Corporation). NEAT1-WT or NEAT1-Mut was co-transfected with miR-31-5p mimic or NC-mimics into cell with Lipofectamine 3000 (Invitrogen, CA, USA). POU2F1-WT or POU2F1-Mut was with miR-31-5p mimic or NC-mimic into cell. After 48-h transfection, a dual-luciferase reporter assay system (Promega, Shanghai, China) was used to explore the luciferase activity. Renilla signals were used to normalize luciferase activity.

### Statistical Analysis

All experiments in this study were repeated at least two or three times and average values of three experiments were presented as the mean standard deviation (SD) calculated by STDEV formula in Excel. The significance of all data was estimated by a Tukey’s multiple-comparison test in the ANOVA analysis using the SigmaStat 3.5 software. Importantly, statistical significance was accepted when *P* < 0.05.

## RESULTS

### NEAT1 Is Increased in LPS-Induced Sepsis

**Fig. 1:**
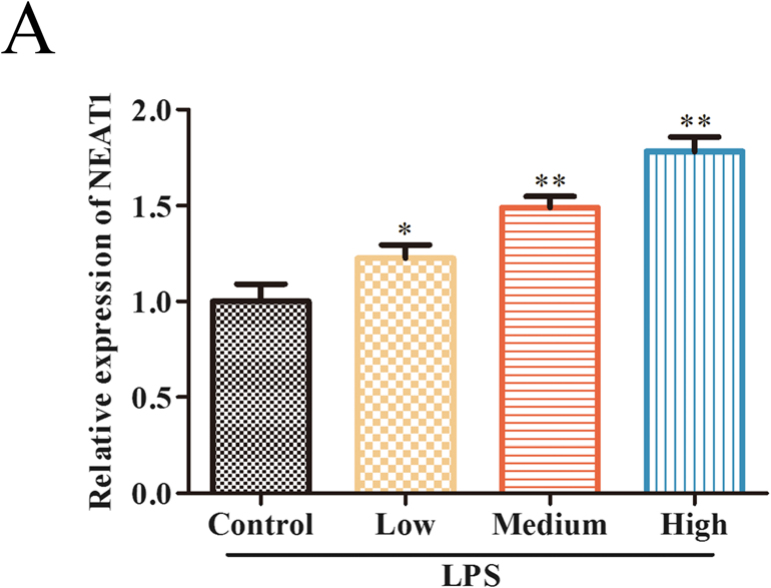
NEAT1 is increased in LPS-induced sepsis. NEAT1 expression in LPS-induced RAW264.7 cells assessed by qRT-PCR. Data were represented as mean ± SD. *P < 0.05, **P < 0.01 vs control

LPS, an important component of Gram-negative bacteria, was the main cause of sepsis [19], which resulted in the inflammatory cascade [20]. In our study, we used different concentrations of LPS (low: 0.01 μg/mL; medium: 0.5 μg/mL; high: 1.0 μg/mL) to treat RAW 264.7 cells and qRT-PCR was performed to examine the expression of NEAT1. As shown in Fig. 1, qRT-PCR illustrated that LPS dose-dependently induced the upregulation of NEAT1 in RAW 264.7 cells, and observed the maximal functions of LPS at the concentration of 1.0 μg/mL.

### Knockdown of NEAT1 Inhibits LPS-Induced Inflammatory Response and Apoptosis in RAW264.7 Cells

**Fig. 2:**
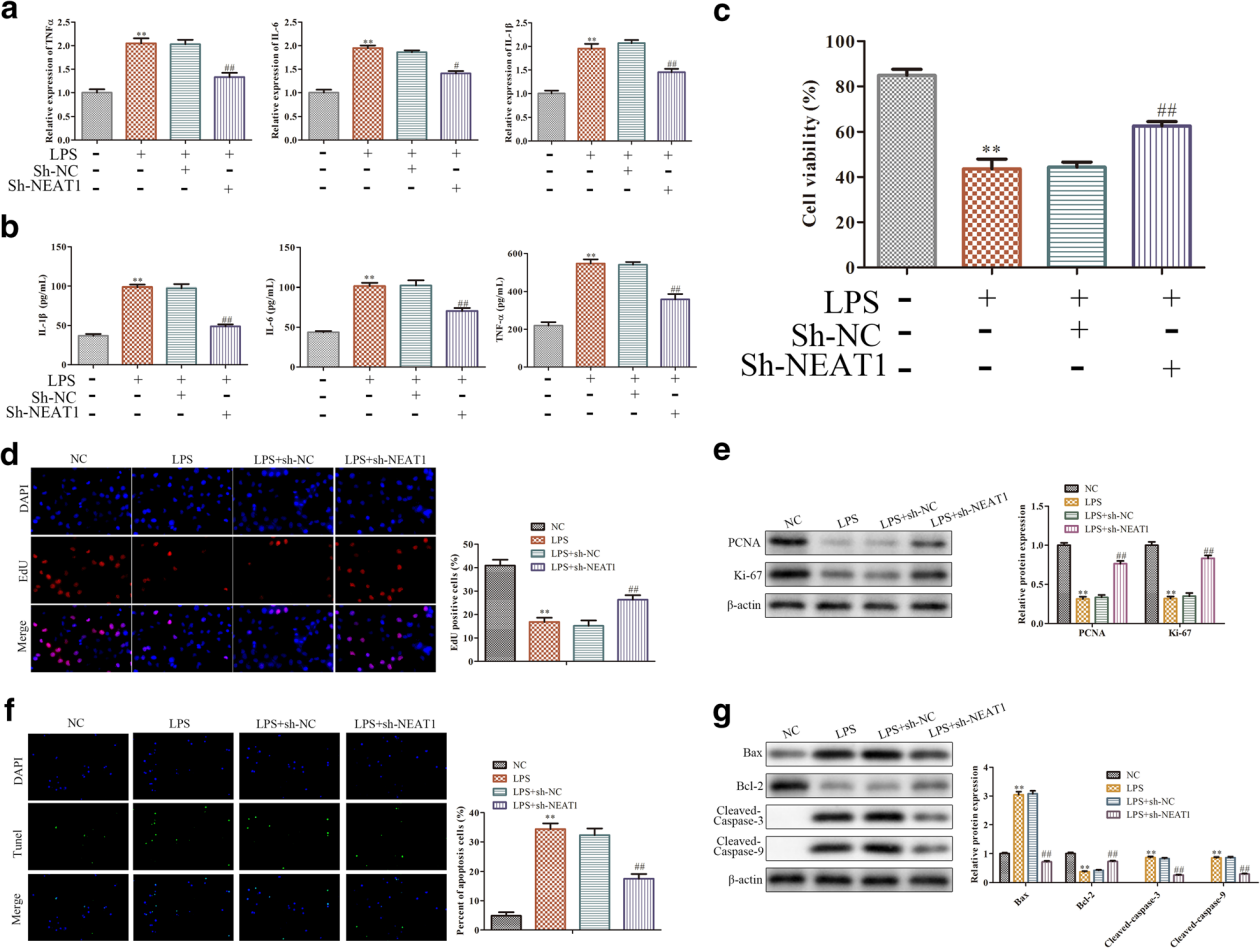
Knockdown of NEAT1 inhibits LPS-induced inflammatory response and apoptosis in RAW264.7 cells. RAW264.7 cells were transfected with sh-NEAT1 or its corresponding control before LPS stimulation. **a** The level of TNF-α, IL-6, and IL-1β measured by qRT-PCR. **b** The concentrations of TNF-α, IL-6, and IL-1β detected by ELISA assay. **c** The cell viability checked by CCK-8 assay. **d** The cell proliferation assessed by EDU staining. **e** The expression levels of PCNA and Ki-67 detected by western blot analysis. **f** The cell apoptosis estimated by TUNEL staining. **g** The expression of Bax, Bcl-2, cleaved-Caspase 3, and cleaved-Caspase 9 evaluated by western blot analysis. Data were represented as mean ± SD. **P < 0.01 vs control, ^#^*P* < 0.05, ^##^*P* < 0.01 vs LPS

To explore the roles of NEAT1 in the inflammatory response of sepsis, RAW 264.7 cells were transfected with sh-NEAT1 and corresponding control group. We firstly detected the content of inflammatory cytokines by Q-PCR and ELISA assay and the data showed that LPS significantly upregulated the level of TNF-α, IL-6, and IL-1β in RAW 264.7 cells, while sh-NEAT1 reversed these effects of LPS on inflammatory cytokines (Fig. 2a, b). Next, CCK-8 assay assessed the cell viability of LPS-stimulated RAW264.7 cells. The cell viability was markedly inhibited by LPS, whereas sh-NEAT1 inhibited LPS-induced cell viability reduction (Fig. 2c). Afterwards, EDU staining was performed and the results illustrated that knockdown of NEAT1 clearly increased the percentage of EDU-positive signals in LPS-induced RAW264.7 cells (Fig. 2d), suggesting that NEAT1 was involved in the RAW264.7 cell proliferation. Likewise, the expression of proliferation-related protein (PCNA, Ki-67) was decreased by LPS compared with control group, while NEAT1 silencing reversed the phenomenon caused by LPS (Fig. 2e). In addition, TUNEL staining showed that NEAT1 silencing inhibited the apoptosis of LPS-induced RAW264.7 cells (Fig. 2f). On the other hand, LPS-induced upregulation of apoptosis-related proteins (Bax, cleaved-Caspase 3, and cleaved-Caspase 9) and downregulation of Bcl-2 protein were rescued by NEAT1 knockdown (Fig. 2g). Collectedly, these results demonstrated that knockdown of NEAT1 exerted an inhibition effect on inflammatory response and apoptosis of LPS-induced sepsis in RAW264.7 cells.

### NEAT1 Functions as a Molecular Sponge for miR-31-5p

**Fig. 3:**
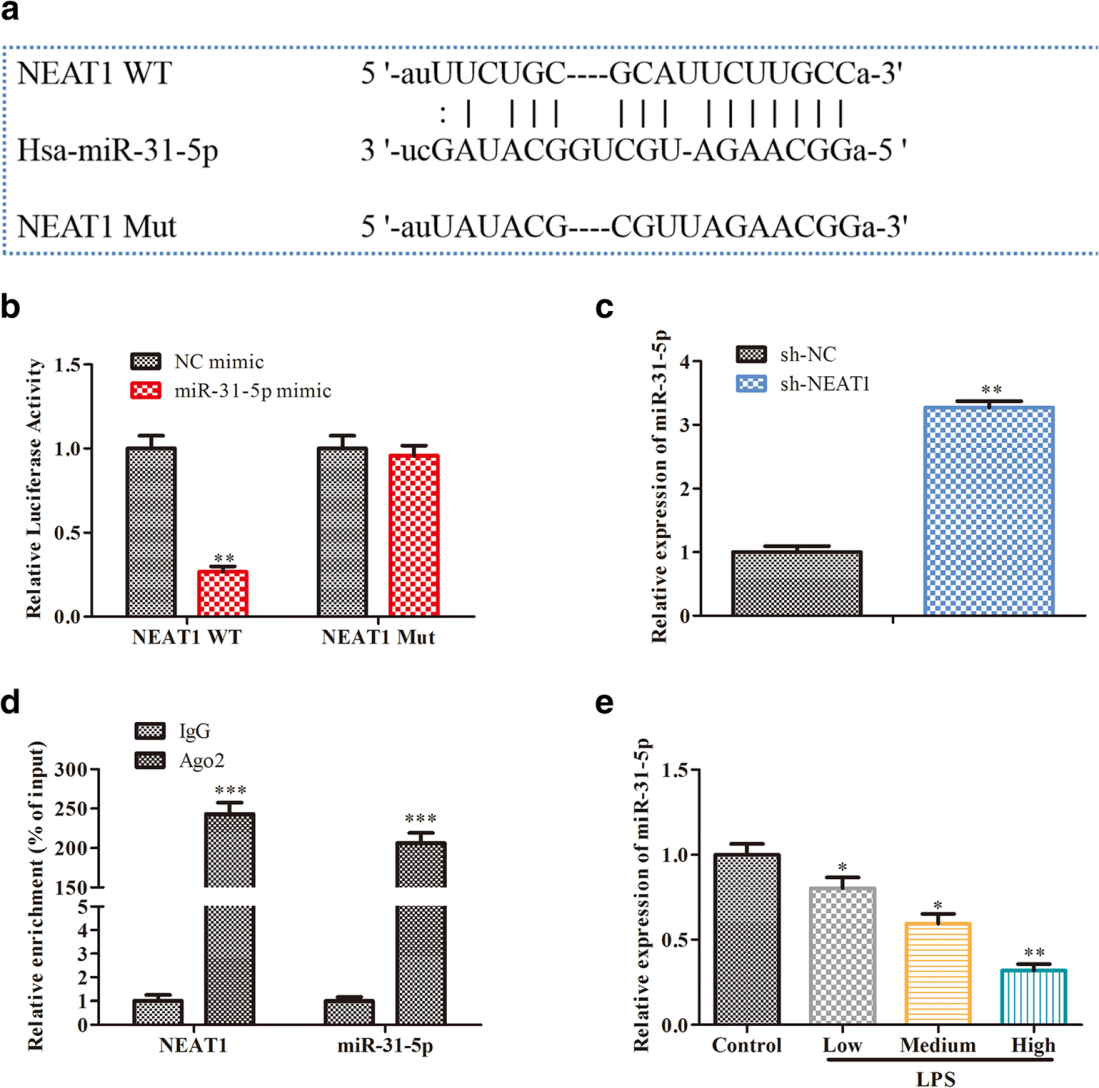
NEAT1 functions as a molecular sponge for miR-31-5p. **a** The binding sites between NEAT1 and miR-31-5p predicted using ENCORI, **P < 0.01 vs NC-mimic. **b** The interaction between NEAT1 and miR-31-5p confirmed by the dual-luciferase reporter assay, **P < 0.01 vs sh-NC. **c** The expression of miR-31-5p detected by qRT-PCR. **d** The interaction between NEAT1 and miR-31-5p confirmed by RIP assay, ***P < 0.001 vs IgG. **e** The expression of miR-31-5p in LPS-induced RAW264.7 cells assessed by qRT-PCR, *P < 0.05, **P < 0.01 vs control. Data were represented as mean ± SD

Increasing evidences reported that lncRNAs regulated the development and occurrence of sepsis by targeting miRNAs [21, 22]. In our study, ENCORI, a prediction software, was used to predict the target miRNA of NEAT1, and we identified miR-31-5p as a potential target, since it is involved in regulating inflammatory responses. We found that there were binding sites between NEAT and miR-31-5p (Fig. 3a). Furthermore, the luciferase activity of NEAT1 WT declined due to miR-31-5p mimic in RAW264.7 cells, while there were no significant effects on NEAT1-Mut (Fig. 3b). Subsequently, qRT-PCR analysis demonstrated that NEAT1 silencing greatly increased miR-31-5p expression (Fig. 3c). Interestingly, RIP experiment proved the binding relationships between NEAT1 and miR-31-5p (Fig. 3d). In addition, we found that LPS inhibited the expression of miR-31-5p in RAW 264.7 cells in a dose-dependent manner (Fig. 3e). All in all, these data demonstrated that NEAT1 regulated the progression of sepsis via sponging miR-31-5p.

### MiR-31-5p Regulates LPS-Induced Inflammatory Responses

**Fig. 4:**
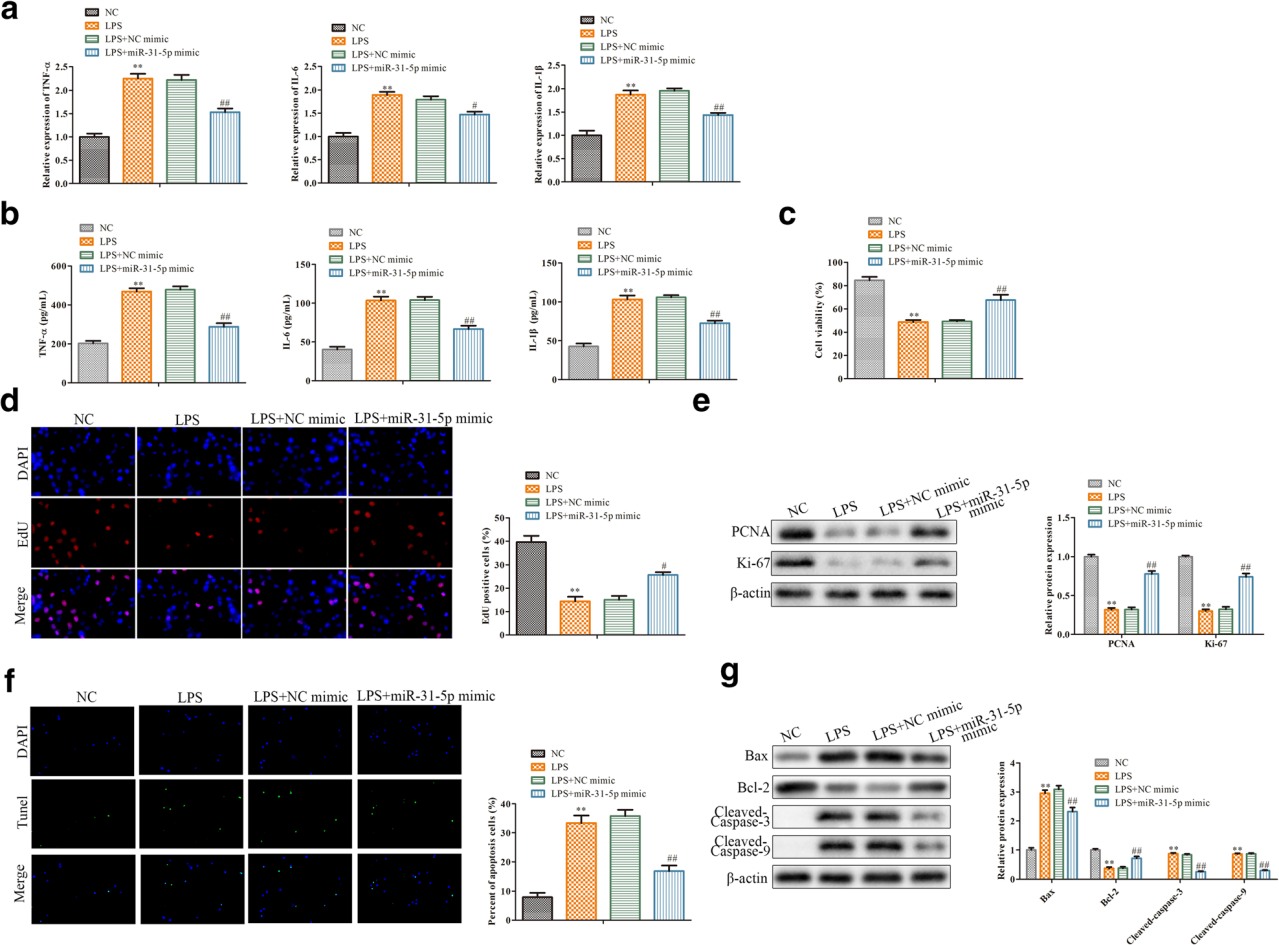
MiR-31-5p regulates LPS-induced inflammatory responses. RAW264.7 cells were transfected with miR-31-5p mimic or its corresponding control before LPS stimulation. **a** The level of TNF-α, IL-6, and IL-1β measured by qRT-PCR. **b** The concentrations of TNF-α, IL-6, and IL-1β detected by ELISA assay. **c** The cell viability checked by CCK-8 assay. **d** The cell proliferation assessed by EDU staining. **e** The expression levels of PCNA and Ki-67 detected by western blot analysis. **f** The cell apoptosis estimated by TUNEL staining. **g** The expression of Bax, Bcl-2, cleaved-Caspase 3, and cleaved-Caspase 9 evaluated by western blot analysis. Data were represented as mean ± SD. **P < 0.01 vs NC, ^*#*^*P* < 0.05, ^*##*^*P* < 0.01 vs LPS

Next, we investigated the role of miR-31-5p in the progression of sepsis. Firstly, LPS-induced RAW264.7 cells were transfected with miR-31-5p mimic and corresponding control. As shown in Fig. 4a and b, upregulation of miR-31-5p obviously inhibited LPS-induced inflammatory factor (TNF-α, IL-6, and IL-1β) upregulation. Thereafter, CCK-8 assay indicated that miR-31-5p overexpression increased the cell viability compared with LPS group (Fig. 4c). To figure out the roles of miR-31-5p on cell proliferation, EDU staining was performed and the results showed that miR-31-5p mimic greatly reversed the reduction of EDU-positive cells caused by LPS (Fig. 4d). Meanwhile, western blot analysis elucidated that overexpression of miR-31-5p promoted PCNA and Ki-67 expression compared with the LPS-treated group (Fig. 4e). In addition, we found that miR-31-5p overexpression overturned LPS-induced cell apoptosis by TUNEL staining (Fig. 4f), and reversed LPS-induced Bax, cleaved-Caspase 3, cleaved-Caspase 9 upregulation, and Bcl-2 downregulation (Fig. 4g). All together, these results confirmed that miR-31-5p had negative effect on sepsis progression.

### MiR-31-5p Directly Interacts with POU2F1

**Fig. 5:**
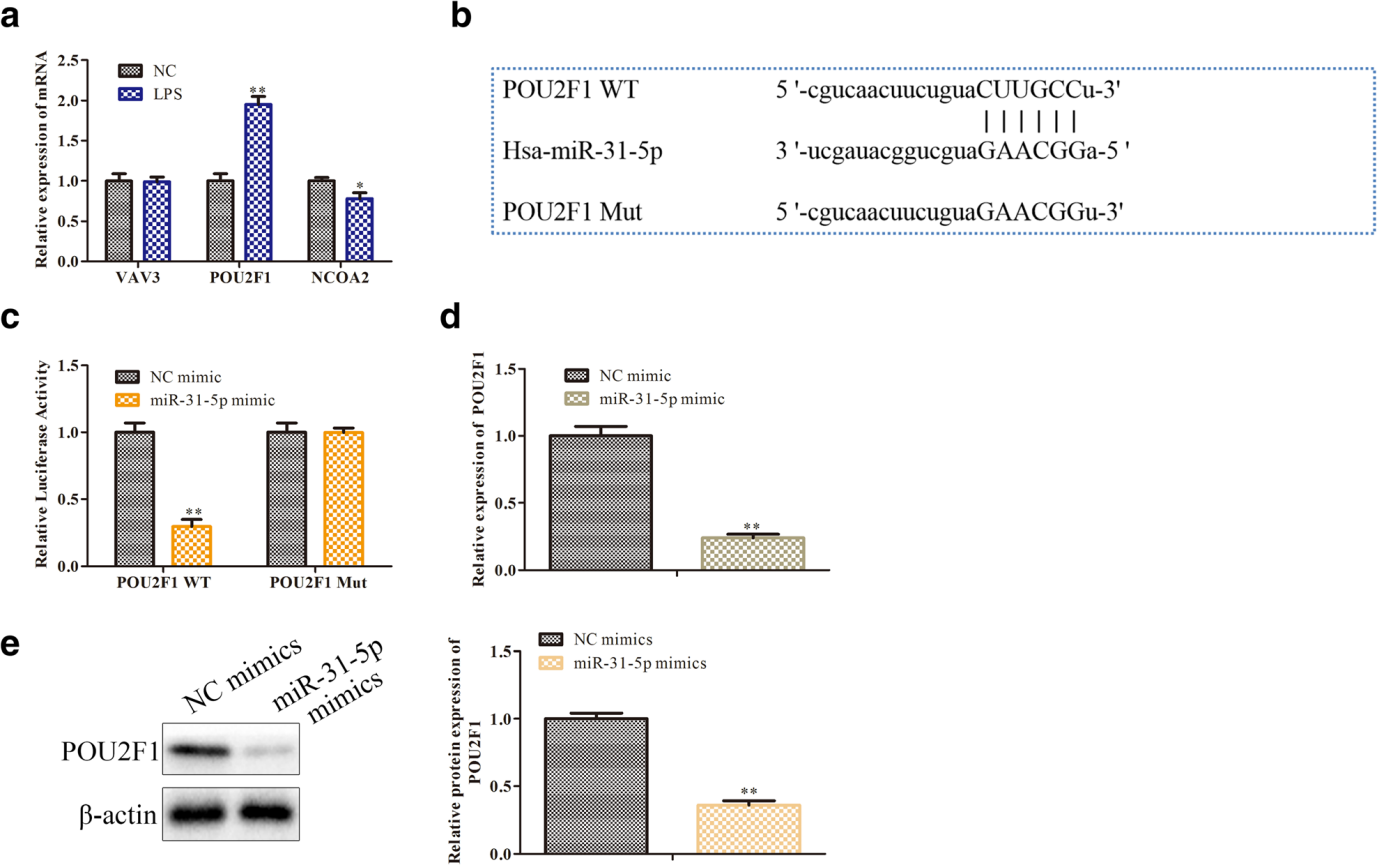
MiR-31-5p directly interacts with POU2F1. **a** The target genes predicted by miRwalk, ENCOR, and TargetScan, *P* < 0.05, **P < 0.01 vs NC. **b** The binding sites between miR-31-5p and POU2F1. **c** The interaction between miR-31-5p and POU2F1 confirmed by the dual-luciferase reporter assay. **d** The mRNA level of POU2F1 measured by qRT-PCR. **e** The expression of POU2F1 detected by western blot analysis. Data were represented as mean ± SD. **P < 0.01 vs NC-mimic

To probe the underlying molecule mechanism of miR-31-5p on sepsis progression, we used miRwalk, ENCORI, and TargetScan prediction software to predict the target gen of miR-31-5p determined POU2F1 since it exerts major roles in sepsis progression (Fig. 5a). We found that there existed binding sites between miR-31-5p and POU2F1 (Fig. 5b). To confirm the relationship between miR-31-5p and POU2F1, the luciferase reporter assay was performed. As shown in Fig. 5c, the luciferase activity significantly decreased following co-transfection with POU2F1-WT and miR-31-5p mimics, compared with co-transfection with POU2F1-WT and NC-mimics, while no evident change was observed in cells transfected with a mutant POU2F1 binding sequence. Besides, miR-31-5p overexpression clearly inhibited the mRNA and protein expression levels of POU2F1 (Fig. 2d, e). Altogether, these results indicated that POU2F1 was a target gene of miR-31-5p.

### NEAT1 Regulates the LPS-Induced Inflammatory Response via miR-31-5p/POU2F1 Axis

**Fig. 6:**
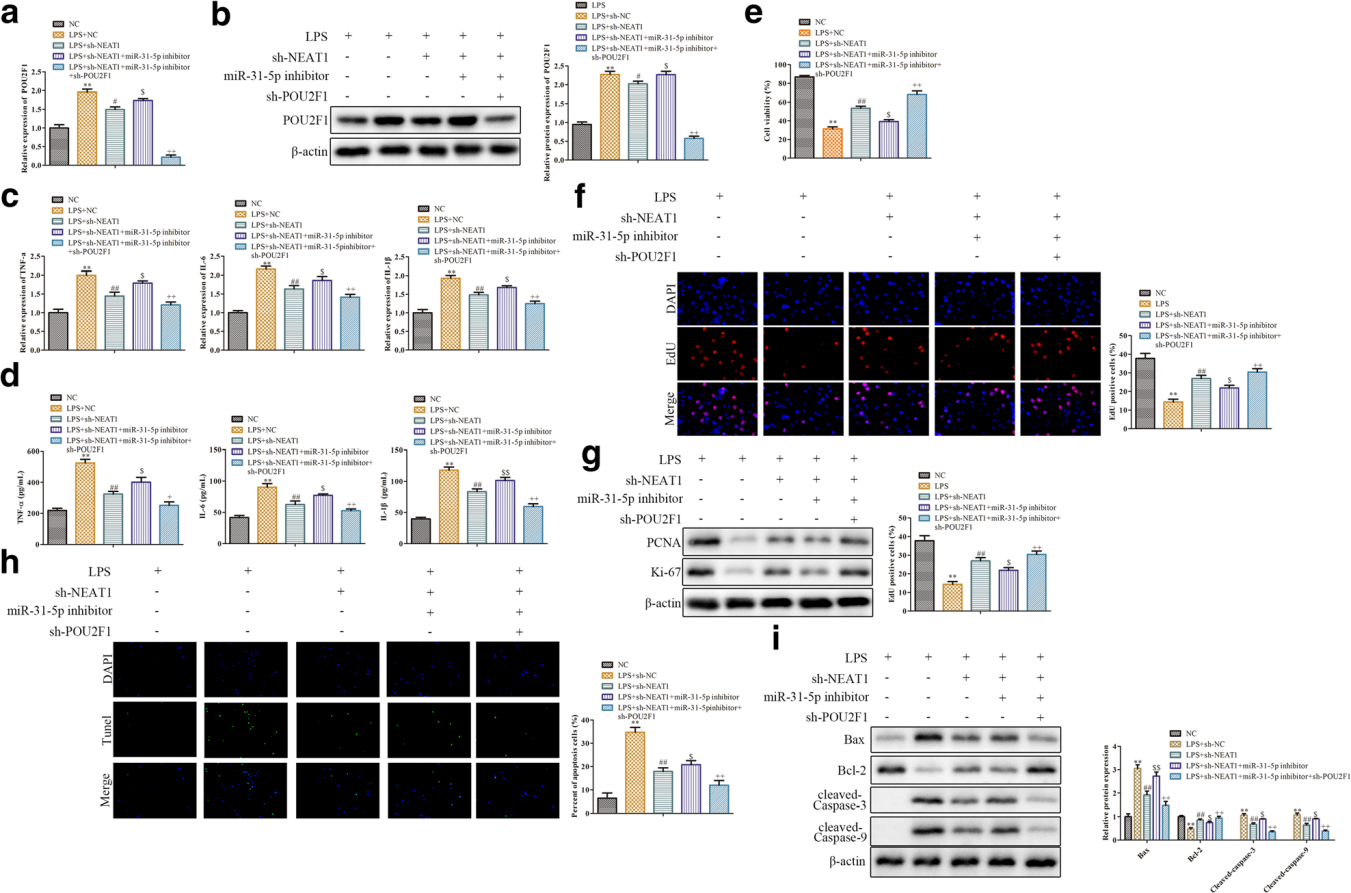
NEAT1 regulates the LPS-induced inflammatory response via miR-31-5p/POU2F1 axis. **a** The mRNA level of POU2F1 measured by qRT-PCR. **b** The expression of POU2F1 detected by western blot analysis. **c** The level of TNF-α, IL-6, and IL-1β measured by qRT-PCR. **d** The concentrations of TNF-α, IL-6, and IL-1β detected by ELISA assay. **e** The cell viability checked by CCK-8 assay. **f** The cell proliferation assessed by EDU staining. **g** The expression levels of PCNA and Ki-67 detected by western blot analysis. **h** The cell apoptosis estimated by TUNEL staining. **i** The expression of Bax, Bcl-2, cleaved-Caspase 3, and cleaved-Caspase 9 evaluated by western blot analysis. Data were represented as mean ± SD. **P < 0.01 vs NC, ^#^*P* < 0.05, ^##^*P* < 0.01 vs LPS, ^$^*P* < 0.05 vs LPS+sh-NEAT1, ^++^*P* < 0.01 vs LPS+ sh-NEAT1+miR-31-5p

Finally, to ascertain NEAT1 promoted sepsis progression by miR-31-5p/POU2F1 axis, we co-transfected RAW264.7 cells with sh-NEAT1 (or sh-NC), sh-POU2F1, and miR-34b-5p inhibitor before LPS stimulation. qRT-PCR and western blot analysis results demonstrated that in LPS-induced RAW264.7 cells, POU2F1 expression was greatly decreased in sh-POU2F1+miR-31-5p inhibitor+sh-NEAT1 group, when compared with miR-31-5p inhibitor+sh-NEAT1 group (Fig. 6a, b). Moreover, in LPS-induced RAW264.7 cells, miR-31-5p silencing removed the inhibition effect of sh-NEAT1 on the levels of inflammatory factors (TNF-α, IL-6, and IL-1β), whereas reversed by knockdown of POU2F1 (Fig. 6c, d). CCK-8 assay showed that the increased RAW264.7 cell viability by NEAT1 knockdown was partly reversed by miR-31-5p inhibitor, whereas POU2F1 knockdown covered the role of miR-31-5p inhibitor (Fig. 6e). Furthermore, in LPS-induced RAW264.7 cells, we found that miR-31-5p silencing could inhibit sh-NEAT1-induced upregulation of the percentage of EDU-positive cells, which partly eliminated by POU2F1 knockdown (Fig. 6f). Likewise, miR-31-5p silencing markedly reduced the expression of proliferation-related proteins compared with sh-NEAT1 group (Fig. 6g), while POU2F1 knockdown reversed the functions of miR-31-5p inhibitor (Fig. 6g). Furthermore, compared to miR-31-5p inhibitor group, knockdown of POU2F1 significantly inhibited cell apoptosis and the expression of Bax, cleaved-Caspase 9, and cleaved-Caspase 3; meanwhile, it promoted the expression of Bcl-2 (Fig. 6h, i). These results manifested that NEAT1 regulated the LPS-induced inflammatory response via miR-31-5p/POU2F1 axis.

## DISCUSSION

Sepsis is a complex syndrome associated with infection-induced immune, endocrine and metabolic response dysregulated, and multiple organ dysfunction [1]. Multiple studies have shown that the levels of inflammation-related factors in serum are directly proportional to the severity of sepsis, such as TNF-α, IL-6, and IL-1β. Recent research has identified the inflammatory response as key for sepsis therapy. LPS as the pathogen-associated molecular pattern has been reported to exacerbate the inflammatory response processes during sepsis [23]. So, we used LPS to stimulate RAW264.7 cell to induce cell inflammatory models. In this study, our data demonstrated that NEAT1 aggravated LPS-induced inflammatory responses and cell apoptotic activity by targeting miR-31-5p and POU2F1, uncovering the potential therapeutic role of NEAT1 in sepsis-induced inflammatory response.

NEAT, located at chromosome 11q13.1, is first discovered in the brain of rats injected with Japanese encephalitis virus and rabies virus in 2002 [24]. Further studies have found that NEAT1 promotes HIV-1 replication, suggesting that NEAT1 may play a key role in responding to viral infection [25]. In addition, NEAT1 directly acts on the expression of inflammatory factors to participate in immune disorders, and accelerates the inflammatory response [26]. Huang *et al*. [27] detect increased NEAT1 expression in peripheral blood of sepsis patients, which is closely related to high expression of inflammatory factors, increased disease risk, increased severity, and poor prognosis [16], indicating that NEAT1 is associated with immune system dysfunction. Given our *in vitro* results, NEAT1 expression was significantly increased in LPS-induced RAW 264.7 cells. Additionally, knockdown of NEAT1 inhibited apoptosis of LPS-induced cells. The levels of inflammatory cytokines (TNF-α, IL-6, and IL-β) also were decreased by NEAT1 silencing in LPS-induced cells. These results indicated that NEAT1 had positive role in sepsis progression. However, it is still unclear how the upregulation of NEAT1 affects LPS-induced sepsis.

As previously mentioned, lncRNAs often bind to miRNA as endogenous competitive RNA (ceRNA) to exert their function [28]. In our research, we found that miR-31-5p was predicted as the downstream target of NEAT and its expression was negatively correlated with NEAT1. Accumulating reports demonstrate that miR-31-5p exerts dual role in tumor malignancy. For instance, miR-31-5p is overexpression in multiple cancer types, including lung cancer [29] and colorectal cancer [30]. In contrast, it is downregulated in prostate cancer [31] and gastric cancer [32]. In addition, emerging evidence finds that miR-31-5p is involved in inflammatory [33, 34]. However, the role of in sepsis progression has not been reported. Our investigation showed that the increased levels of TNF-α, IL-6, and IL-β in LPS-induced RAW 264.7 were inverted after the infection with miR-31-5p mimic.

We further explored the downstream cascade of NEAT1/miR-31-5p axis involved in sepsis-induced inflammatory response, and found miR-31-5p target gene, POU2F1. POU2F1, a pervasive transcription factor (TF), exerts major roles in the regulation of inflammatory-related gene [35]. In this research, we found that POU2F1 markedly increased in LPS-induced RAW264.7 cells, whereas knockdown of NEAT1 significantly recovered the expression of POU2F1. Moreover, the expression of POU2F1 was negatively regulated by miR-31-5p, but was positively correlated with NEAT1. Further rescue experiment indicated that knockdown of POU2F reversed the positive impact of miR-31-5p silencing on sepsis progression, suggesting the involvement of POU2F1 in sepsis-induced inflammatory response.

**Fig. 7:**
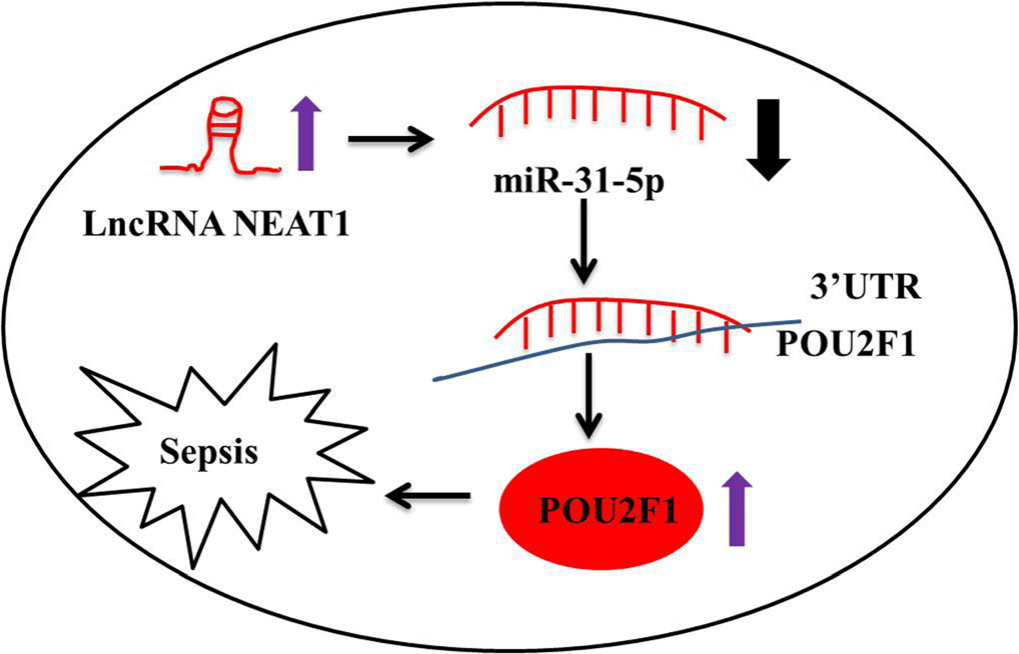
A schematic of the NEAT1 acting as a molecular sponge for miR-31-5p/POU2F1 to modulate inflammatory response

In summary, all these findings demonstrated that NEAT1 promoted inflammatory response in sepsis via the miR-31-5p/POU2F1 axis (Fig. 7), and it provided a novel therapeutic target for sepsis.

## Data Availability

All data generated or analyzed during this study are included in this published article.
